# A new herbal anesthetic agent for common carp (*Cyprinus carpio*) sedation and anesthesia: nutmeg (*Myristica fragrans*) essential oil

**DOI:** 10.3389/fvets.2024.1477357

**Published:** 2024-10-14

**Authors:** Mert Minaz

**Affiliations:** Department of Aquaculture, Faculty of Fisheries, Recep Tayyip Erdoğan University, Rize, Turkey

**Keywords:** DNA damage, fish, hematology, herbal anesthetic agent, histology

## Abstract

In aquaculture, interest in natural essential oils is increasing alongside synthetic anesthetic agents. In this context, the anesthetic efficacy of nutmeg essential oil, which had not been previously tested, was investigated in common carp (*Cyprinus carpio*). The study, conducted using three different concentrations (800 μL/L “LC”, 1,200 μL/L “MC”, and 1,400 μL/L “HC”), found that induction times were <3 min for MC and HC, while LC had a longer induction time, exceeding acceptable levels. Within the first 4 h, white blood cells, red blood cells, hemoglobin, and hematocrit levels increased to >5 10^3^/μL, >1 10^6^/μL, >6 g/dL, and >12%, respectively. However, they returned to control levels after 8 h. Histological signs were more severe with higher concentrations, and necrosis was only observed in the HC group. Alkaline comet assay results showed DNA migration only in the HC group. According to the PROMETHEE multi-criteria decision-making model, the LC concentration is suitable for sedation, while the MC concentration should be used for deep anesthesia. The current study demonstrates that nutmeg essential oil can be used as an alternative to commercial synthetic anesthetic agents.

## 1 Introduction

In aquaculture, fish undergo routine procedures including fish-grading, transportation, vaccination, tagging, and surgery. These activities elevate stress levels in fish, significantly affecting their physiology and survival (1, 2). Stress negatively impacts fish physiology, causing slower growth, suppressed immune function, and inhibited reproduction (3, 4). Reduced fish welfare directly affects fish health and growth, leading to economic losses in the aquaculture industry (5). Thus, anesthetic agents are widely used in aquaculture to reduce stress and eliminate pain sensitivity. Effective sedation and anesthesia can help mitigate the negative effects associated with the hypothalamic-pituitary-interrenal (HPI) axis, which is central to physiological stress responses (6). Beyond safeguarding fish welfare, anesthesia is employed to immobilize fish during demanding procedures such as blood sampling and spawning of large broodstock. This practice not only alleviates the workload but also minimizes the risk of potential injuries. Additionally, anesthetics are often used during fish transportation to decrease metabolic activity and prevent mechanical damage.

The most commonly used anesthetics in aquaculture include Tricaine methanesulfonate (MS-222), Benzocaine, Quinaldine, 2-Phenoxyethanol (2-PE), Metomidate, Aqui-S, and clove oil (8, 83). Due to the negative effects of synthetic anesthetics on fish and their potential residues affecting humans, researchers in recent years have been investigating the anesthetic properties of plant-based essential oils (7, 9–11). The use of natural essential oils as anesthetic agents for fish is gaining increasing attention due to their potential benefits over synthetic alternatives (12). Synthetic anesthetics, while effective, can leave harmful residues that affect both aquatic environments and human health. In contrast, essential oils, derived from natural sources such as clove or lavender, offer a more eco-friendly and sustainable option (7, 10). These natural anesthetics are often less toxic, degrade more rapidly in water, and reduce the risk of harmful residues (13). Additionally, essential oils can effectively induce anesthesia while minimizing stress and injury to the fish. Their application not only enhances fish welfare during procedures like handling and transportation but also aligns with the growing emphasis on environmental sustainability and reducing chemical pollutants in aquaculture practices.

As a natural anesthetic, nutmeg, native to the Maluku Islands of Indonesia, is also distributed in India, Sri Lanka, South Africa, and the United States (14, 15). While nutmeg is primarily used as a spice (16), it is also extracted to produce essential oil, which possesses antioxidant, antimicrobial, anti-inflammatory, anticancer, and aphrodisiac properties (17–20). However, while there is one study on the potential of nutmeg powder as an anesthetic agent (21), there have been no studies conducted on its essential oil for this purpose. The reports on the narcotic use of nutmeg (22, 23) have led to the idea that this substance might induce anesthesia effects when used in appropriate concentrations. Myristicin, an important component of nutmeg, can exhibit adverse effects such as hallucinations, tachycardia, drowsiness, numbness, and blurred vision (24). Therefore, as a first step, it is necessary to investigate the potential effects of myristicin to understand how the ingestion of large amounts of nutmeg could impact patients in the field of anesthesia (25).

The effectiveness of an anesthetic agent is evaluated based on several factors, including induction time, physiological effects, and cost analysis. Responses to anesthetics can vary among different fish species, so the efficacy of an anesthetic may differ from one species to another. The common carp (*Cyprinus carpio*), a warm-water fish with a low trophic level, is one of the most significant species in the aquaculture industry (26). Due to its high adaptability to environmental conditions, it is considered a valuable fish for aquaculture. In many European countries, common carp constitutes more than 80% of total fish production (27). Therefore, to increase the acceptability of a previously unused natural anesthetic agent, it is essential to focus on a significant species in aquaculture. In light of this motivation, the current study focuses on the applicability and effects of nutmeg, a naturally occurring agent with anesthetic potential, on common carp. In addition, it aims to determine optimal concentration of the nutmeg oil by using a multi-criteria decision-making method (PROMETHEE).

## 2 Material and method

### 2.1 Nutmeg (*Myristica fragrans*)

The nutmeg (*Myristica fragrans*) essential oil was purchased from a commercial corporation (Doalinn, Türkiye). Since it does not dissolve in water on its own, it was dissolved in a 1:10 ratio using 96% ethanol. The content of nutmeg oil is presented in Table 1. Analyses were performed on a GC–MS (Shimadzu QP2010 Ultra) device with a 30 m 5-Ms column. The temperatures of transfer line and ion source were 280 and 275°C, respectively. Qualitative analysis was done using NIST and Wiley libraries integrated in the device. The GC-MS analysis was carried out as an outsourced service at the Central Research Laboratory Application and Research Center in Recep Tayyip Erdoğan University. Accordingly, α-pinene, β-pinene, and sabinene are the main components (>10%) of nutmeg essential oil.

**Table 1:** Composition of nutmeg essential oil.

**Peak**	**Constituents**	**Height (%)**
1	α Pinene	17.175
2	β Pinene	10.648
3	Sabinene	20.745
4	Δ-3-Carene	2.018
5	Myrcene	2.171
6	α Phellandrene	1.216
7	A Terpinene	4.312
8	Limonene	5.102
9	β Phellandrene	3.071
10	ɤ Terpinene	6.689
11	Cymene	2.437
12	α Terpinolene	2.141
13	4-Terpineol	8.085
14	Safrole	1.479
15	Mthyleugenol	1.034
16	Elemicine	3.537
17	Myristicin	3.410
18	Others <1.000%	4.730
		100

### 2.2 Experimental trial

The current study was approved by the Ethical Committee of Recep Tayyip Erdoğan University, Türkiye (Decision No: 2024/05). The trials were conducted at the Recep Tayyip Erdoğan University Aquaculture Research and Application Center. In the study, the fish were exposed to three different anesthetic concentrations of 800 (LC), 1,200 (MC), and 1,400 (HC) μL/L, respectively. The concentrations of nutmeg oil were determined based on induction and recovery times as a result of a preliminary study. As in our previous study, ethanol was added to the control group at the highest concentration, and this is considered negligible as an anesthetic (7). A total of 120 fish (46.8 ± 4.9 g) were used, with each group consisting of three replicates and ten fish per replicate. For adaptation, the fish were placed in the trial tanks (12 different tanks with 50 L volume) 7 days before the study and were not fed during the last 48 h. The adaptation system was a flowthrough system with 7.2 ± 0.3 pH and 8 ± 0.9 mg/L DO. The water source for the study was groundwater with a constant temperature of 18°C. Induction time was determined by observing reactions such as a complete lack of response to stimuli (handling), abnormal operculum movements, and loss of balance (28). The fish behavioral responses to anesthesia were monitored over a 7-min period. Recovery was defined as the moment when the fish began to swim steadily and responded to stimuli. After induction, the anesthetized fish were immediately placed in 500 L tanks with vigorous aeration using an air pump (DO 9.8 ± 0.7 mg/L) for recovery. Induction and recovery procedures were performed individually for each fish. To eliminate observer bias, both the recovery and induction times were recorded by a single observer with a stopwatch.

### 2.3 Hematological examination

For hematological studies, blood samples were taken from the anesthetized fish at 2, 4, and 8 h after initial anesthesia, without additional anesthetic operation. Samples were collected from the control group without anesthesia. Five fish were randomly chosen (the fish from which blood was drawn were excluded from the study) and blood was collected from caudal vein with a 1 mL syringe. Then, blood samples were placed in EDTA K3 tubes to prevent clotting. Red blood cells (RBC), white blood cells (WBC), hemoglobin (HGB), and hematocrit (HCT) were measured with an automatic hematological analyzer (Prokan-6800VET, Shenzhen, China). The instrument was calibrated with blank blood samples of the common carp before the study (29).

### 2.4 Histological evaluation

Only gill tissues were considered for histological examination. Three fish (the fish from which blood was drawn were excluded from the study), exposed to the anesthesia, were euthanized (mechanical stunning), and gill tissues (second lamella) were later taken. Control fish were euthanized with mechanical stunning. Tissues were fixed in 10% neutral buffer formalin for 2 days and then transferred to 50% ethanol. Gills were subjected to alcohol series and placed into the liquid paraffin at 65°C for 12 h. Then, tissues were blocked in the paraffin and sectioned at a thickness of 5 μm using a microtome. Preparations were transferred at 65°C to remove paraffin and subjected again to ethanol and xylene series. Afterwards, samples were stained with hematoxylin and eosin and covered with Entellan^®^ (MERCK/107961.0500) and cover-slip. Histological changes were observed with light microscope and imaged (30).

### 2.5 Alkaline comet assay

A maximum of 10 μL blood sample was taken from the caudal vein of both anesthetized and control fish. An alkaline (pH > 13) environment was provided for erythrocyte DNA damage assessment (31). Blood samples taken from three fish (from each group) were mixed with 1 mL Phosphate Buffed Saline (PBS-Ca ++, MG ++ free). Subsequently, 15 μL of this cell suspension (~10,000 cells) was taken and mixed with 75 μL of 0.5% low melting point (LMP) agarose. In the further stage, the frosted glass slides were covered with three layers: (1) 1% normal melting point (NMP) agarose, (2) LMP + cell suspension, and (3) 100 μL LMP agarose. These layers were then allowed to solidify at 4°C. The samples were incubated in lysing solution (2.5 m NaCl, 100 mm EDTA, 10 mm Tris, PH 10.0–10.5, 1% Triton X-100 and 10% DMSO) for 8 h to allow for cell lysis. The slides were then placed in a horizontal gel electrophoresis unit containing fresh cold alkaline electrophoresis buffer and cell migration was achieved for 30 min at 25 V and 300 mA. After this step, samples were treated for 10 min with neutralized buffer. Slides were washed with distilled water for 10 min and stained with 0.5 μg/ml ethidium bromide. Distilled water was used to prevent over staining. All procedures up to this stage were carried out at +4°C and in a dark environment. For the positive control, hydrogen peroxide (H_2_O_2_) treatment (three fish) was applied for 10 min. For each sample, three slides were examined under the fluorescent microscope (Leica DMR HC, Germany). In addition, cells were analyzed using Comet ScoreTM 2.0 Software (Tritek Crop., Sumemeruck, VA, USA).

### 2.6 PROMETHEE decision model

The optimal concentration was determined based on a multi-criterion decision-making (MCDM). The Preference Ranking Organization Method for Enrichment (PROMETHEE) model comprises four fundamental steps: (1) investigation of the appropriate anesthetic concentration for common carp, (2) determination of evaluation criteria and weights for each concentration group, (3) scoring the different concentration groups depending on evaluation criteria, and (4) decision on the best anesthetic concentration. Each alternative was weighted according to several evaluation criteria. Each evaluation criterion contains a weight value, and the sum of these values is equal to “1” (32).

According to the MCDM principle, alternatives should be evaluated based on the specified criteria. These criteria are used by the decision-maker when assessing and reaching a conclusion. To determine the optimal anesthetic concentration, evaluation criteria are considered based on feasibility, cost, effectiveness of the anesthetic agent, and the physiological condition of the fish. In the present study, 15 different evaluation criteria were identified. These criteria were categorized under five main headings: (1) induction and recovery times, (2) cost analysis, (3) hematological parameters, (4) histological changes, and (5) DNA comet assay. The weight value for each criterion was determined based on its importance factor through expert opinions. Thus, the weight values were determined by evaluating the responses received from 10 different professionals in the field of aquaculture (32). The evaluation criteria and their weights are presented in Table 2. For cost analysis, one liter of anesthetic was considered to cost 900 USD ($) and was normalized based on the amount of anesthetic used for each fish species. The average value of blood parameters at the eighth hour for the relevant group was evaluated. For histological changes, a five-point Likert scale indicating the severity of lesions was used. The PROMETHEE decision model reaches results in seven steps (Supplementary material 1).

**Table 2:** Evaluation criteria and weighting scale.

**Criteria number**	**Evaluation criteria**	**Weight value**	**Preference function**
C1	Induction time	0.10	Linear
C2	Recovery time	0.10	Linear
C3	Cost	0.10	V-shape
C4	WBC	0.10	Linear
C5	RBC	0.05	V-shape
C6	HGB	0.05	V-shape
C7	HCT	0.05	Linear
C8	Necrosis	0.10	Usual
C9	Hyperplasia	0.05	Level
C10	Hypertrophy	0.05	Level
C11	Head DNA%	0.05	Linear
C12	Tail DNA%	0.05	Linear
C13	Olive moment	0.05	Linear
C14	Tail length	0.05	Linear
C15	Tail moment	0.05	Linear

### 2.7 Statistical analysis

All data are presented as the means ± standard deviation (SD). The Kolmogorov-Smirnov test was conducted to assess the normality of the distribution. Significant differences between groups were determined with One-way ANOVA and Tukey tests. Differences were considered statistically significant when the calculated *p*-value was <0.05. All analyses were performed in SPSS software (Version 23, IBM Corp., Armonk, New York, USA).

## 3 Results

### 3.1 Effectiveness of anesthetic agent

**Figure 1:**
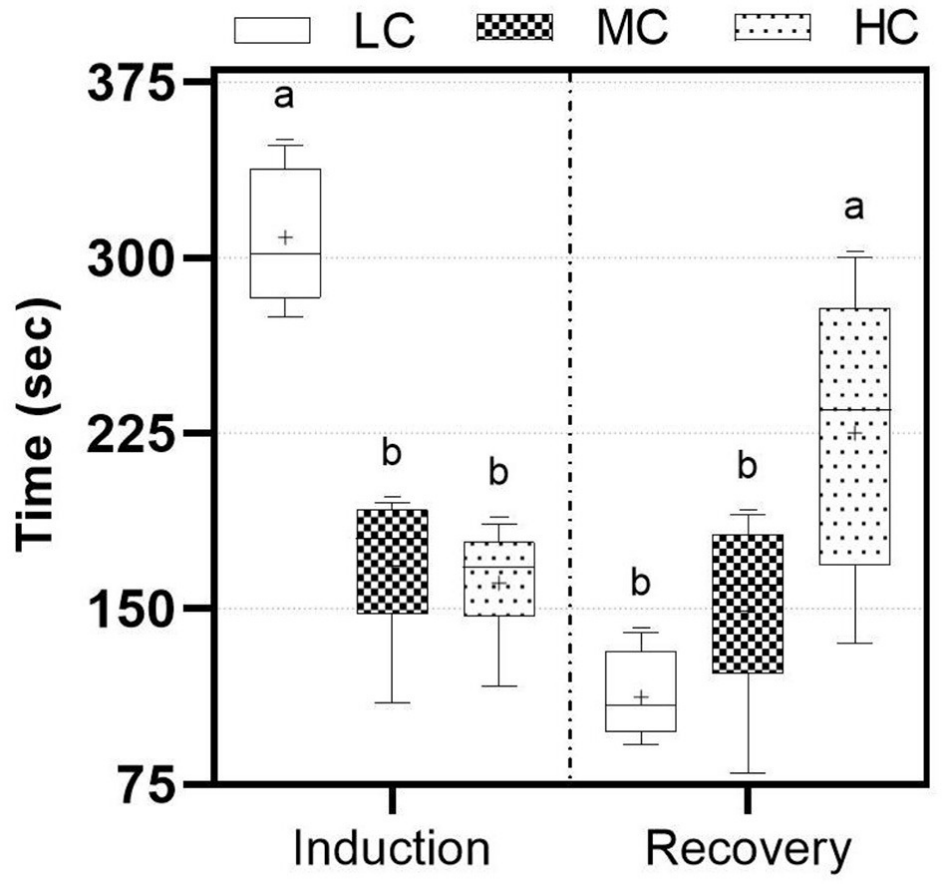
Induction and recovery times according to different nutmeg essential oil concentration. LC, 800 μL/L; MC, 1,200 μL/L; HC, 1,400 μL/L. Lowercase letters represent statistical differences.

The concentration of nutmeg essential oil is significant for induction and recovery times (*p* < 0.05). Fish in the LC group were induced significantly longer, taking more than 5 min (Figure 1). Conversely, fish in the HC group recovered in a significantly longer time (<5 min) compared to other groups.

### 3.2 Hematological results

**Figure 2:**
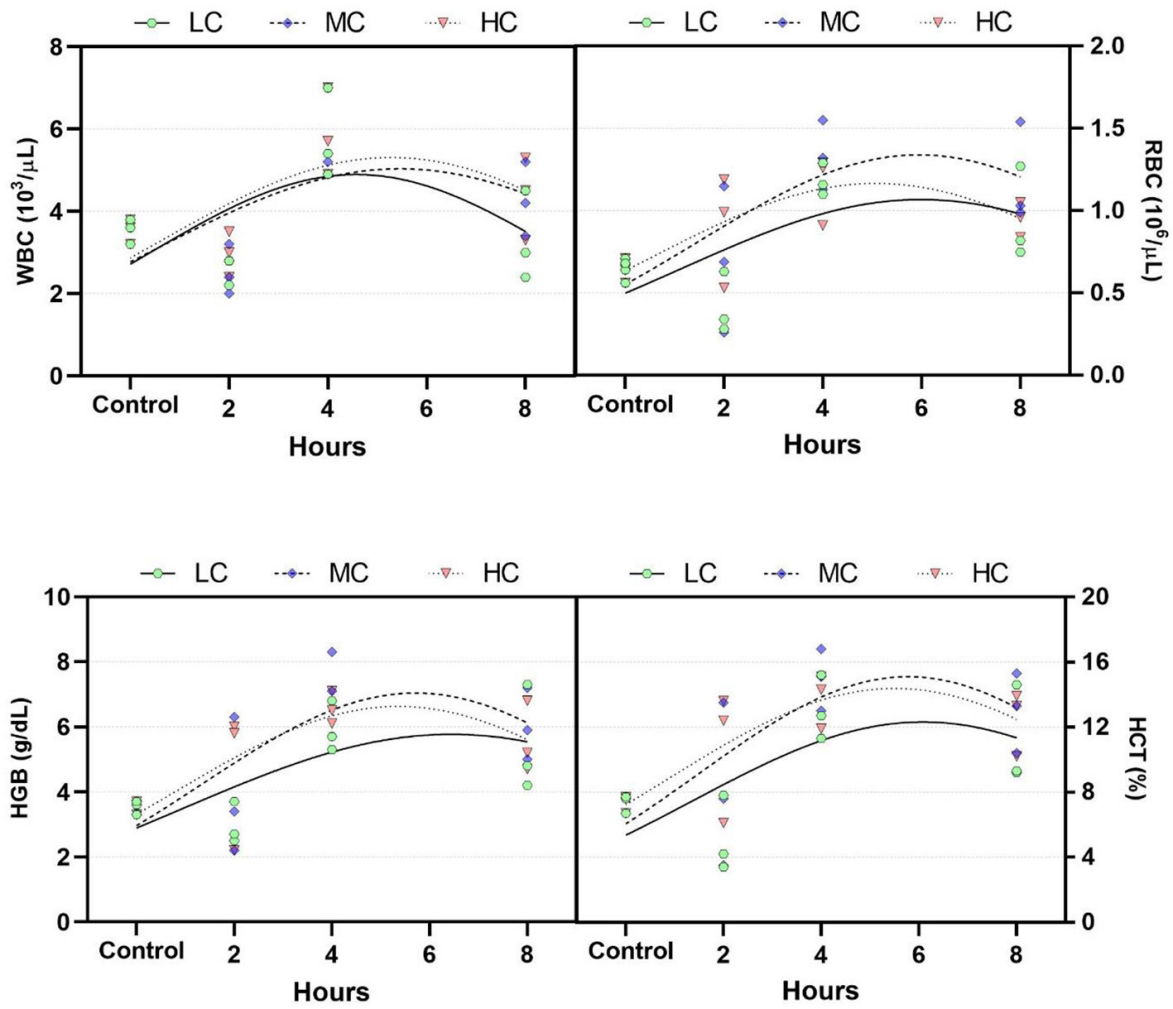
Changes in blood parameters over time. LC, 800 μL/L; MC, 1,200 μL/L; HC, 1,400 μL/L. WBC, white blood counts; LYM, lymphocyte counts; RBC, red blood counts; HGB, hemoglobin concentration.

Blood samples taken at the 2nd, 4th, and 8th hours from anesthetized fish demonstrate the temporal response of the fish's blood parameters (Figure 2). Blood parameters for WBC, RBC, HGB, and HCT increased at the 4th hour and returned to control levels at the 8th hour. The critical peak for all blood parameters was identified between the 5th and 6th hour across all groups. No significant differences in blood parameters were observed at any time points among the concentration groups (*p* > 0.05).

### 3.3 Histological studies

**Figure 3:**
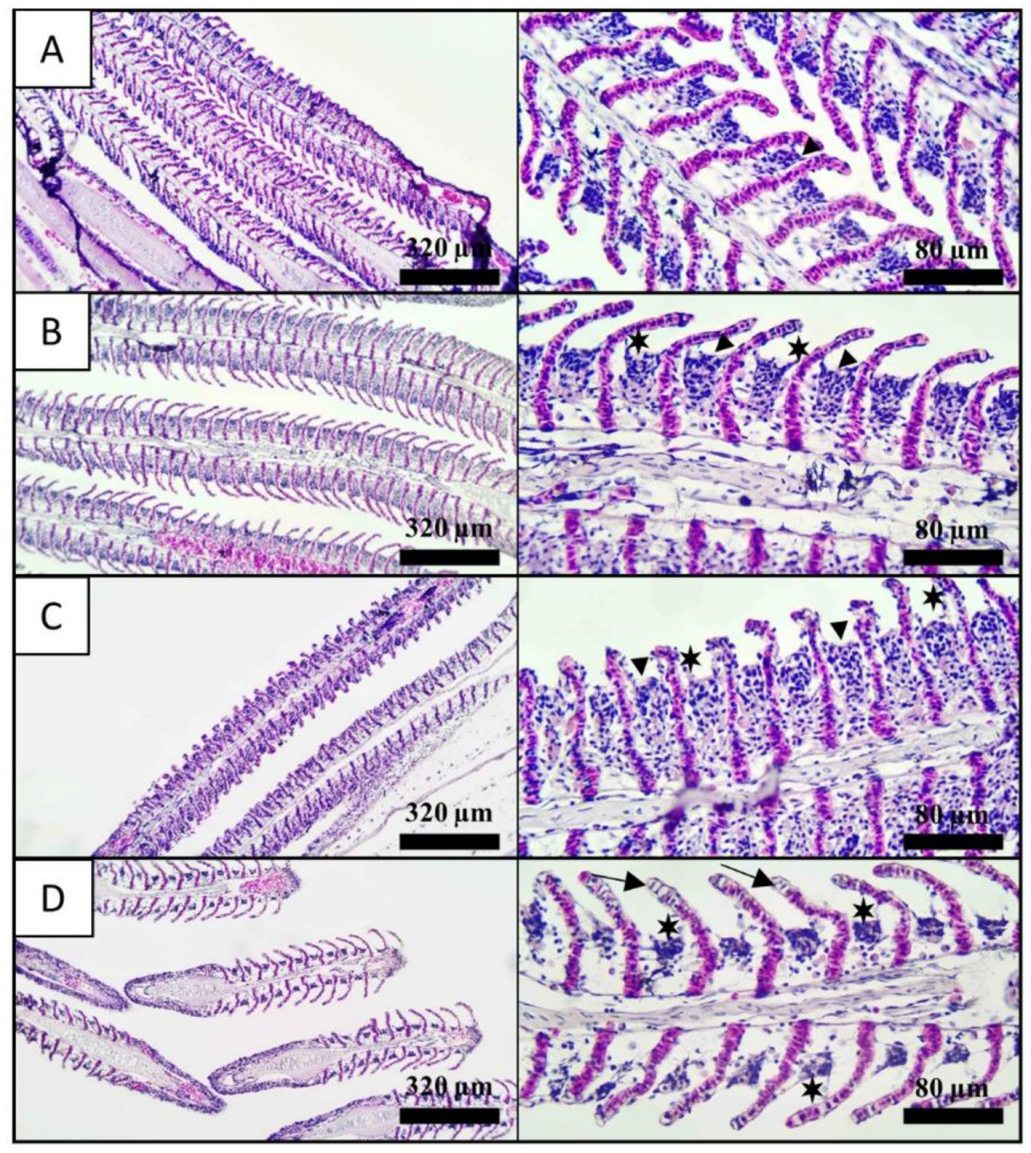
Histological observation of gill tissues exposed nutmeg essential oil with different concentrations. **(A)** Control, **(B)** 800 μL/L, **(C)** 1,200 μL/L, and **(D)** 1,400 μL/L. Arrowhead: hyperplasia, star: epithelial lifting, and arrow: necrosis. Left side and right side in the figure represents 10X and 40X magnification, respectively.

The gill tissues of fish anesthetized with different concentrations of nutmeg essential oil, as well as control fish, were histologically evaluated (Figure 3). Hyperplasia was observed in the gills of control fish. Additionally, hyperplasia and epithelial lifting were noted in the LC and MC groups. In the HC group, epithelial lifting and necrotic cells were prominently observed (Table 3).

**Table 3:** Severity of different histological changes in gill tissues of *C. carpio*.

	**Control**	**LC**	**MC**	**HC**
Hyperplasia	+	+++	++++	+
Epithelial lifting	–	++	++	+++
Necrosis	–	–	+	+++

–, none; +, mild; ++, moderate; +++, severe; ++++, very severe.

### 3.4 DNA damage

**Figure 4:**
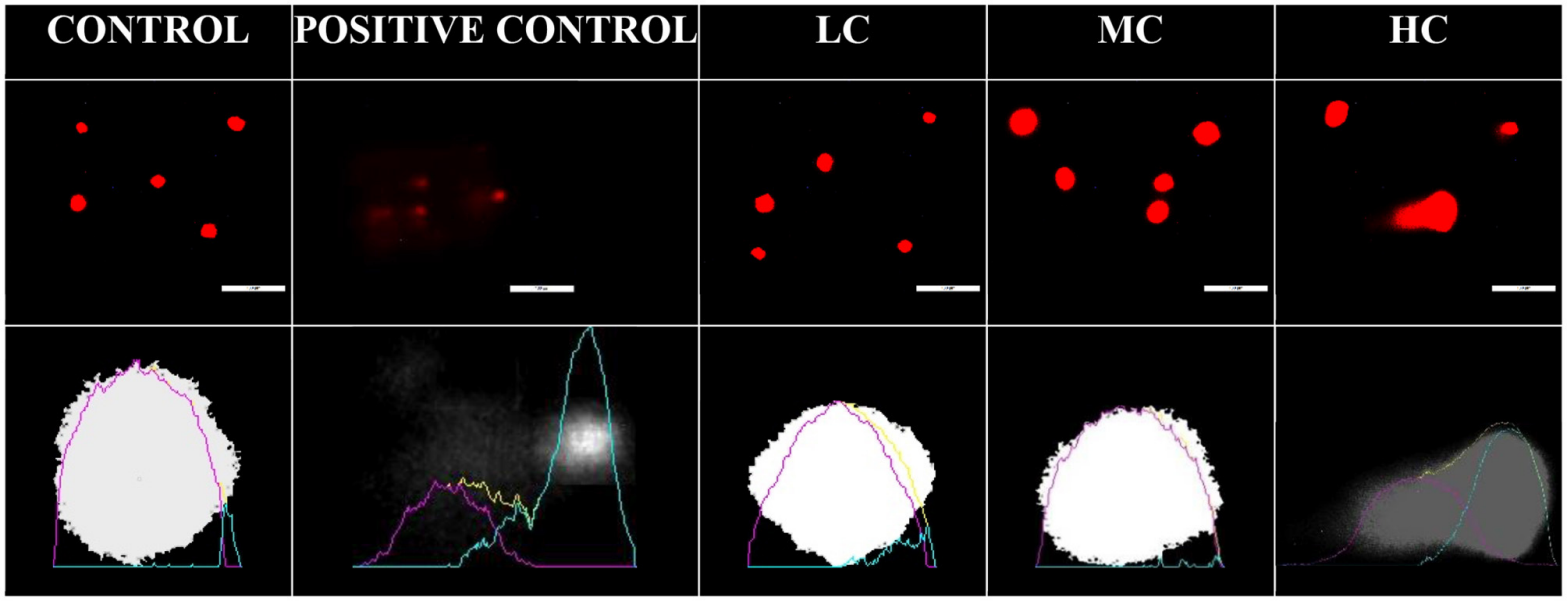
Blood cell DNA migration from control and anesthesia groups. LC, 800 μL/L; MC, 1,200 μL/L; HC, 1,400 μL/L. Positive control was treated with H_2_O_2_ solution.

The potential genotoxic effects of the anesthetic agent were evaluated using the alkaline comet assay method (Figure 4). No DNA migration was observed in the control, LC, and MC groups, whereas DNA damage was notably observed in the blood cells of the HC group. Similar to the positive control treated with H_2_O_2_, the HC group exhibited significantly higher tail DNA%, olive moment, tail length, and tail moment (*p* < 0.01) (Table 4).

**Table 4:** Comet assay parameters for control and anesthesia groups.

	**Head DNA%**	**Tail DNA%**	**Olive moment**	**Tail length**	**Tail moment**
Control	96.7 ± 1.1^a^	3.2 ± 1.1^b^	1.4 ± 0.6^b^	3.5 ± 3.8^b^	0.1 ± 0.05^b^
Positive Control	36.4 ± 12.2^b^	59.9 ± 12.5^a^	38.6 ± 17.7^a^	53.2 ± 23.1^a^	44.8 ± 17.8^a^
LC	96.5 ± 2.6^a^	4.0 ± 3.1^b^	1.3 ± 1.2^b^	1.8 ± 1.9^b^	0.09 ± 0.05^b^
MC	98.9 ± 1.1^a^	1.1 ± 1.0^b^	0.8 ± 0.7^b^	2.4 ± 2.0^b^	0.05 ± 0.04^b^
HC	46.9 ± 21.4^b^	53.5 ± 21.5^a^	51.5 ± 26.8^a^	50.4 ± 24.0^a^	38.2 ± 20.1^a^
F and (*p*) values	38.42 (*p* < 0.01)	35.02 (*p* < 0.01)	14.37 (*p* < 0.01)	16.31 (*p* < 0.01)	17.95 (*p* < 0.01)

LC, 800 μL/L; MC, 1,200 μL/L; HC, 1,400 μL/L. Lowercase letters represent statistical differences.

### 3.5 Multi-criteria decision model

**Figure 5:**
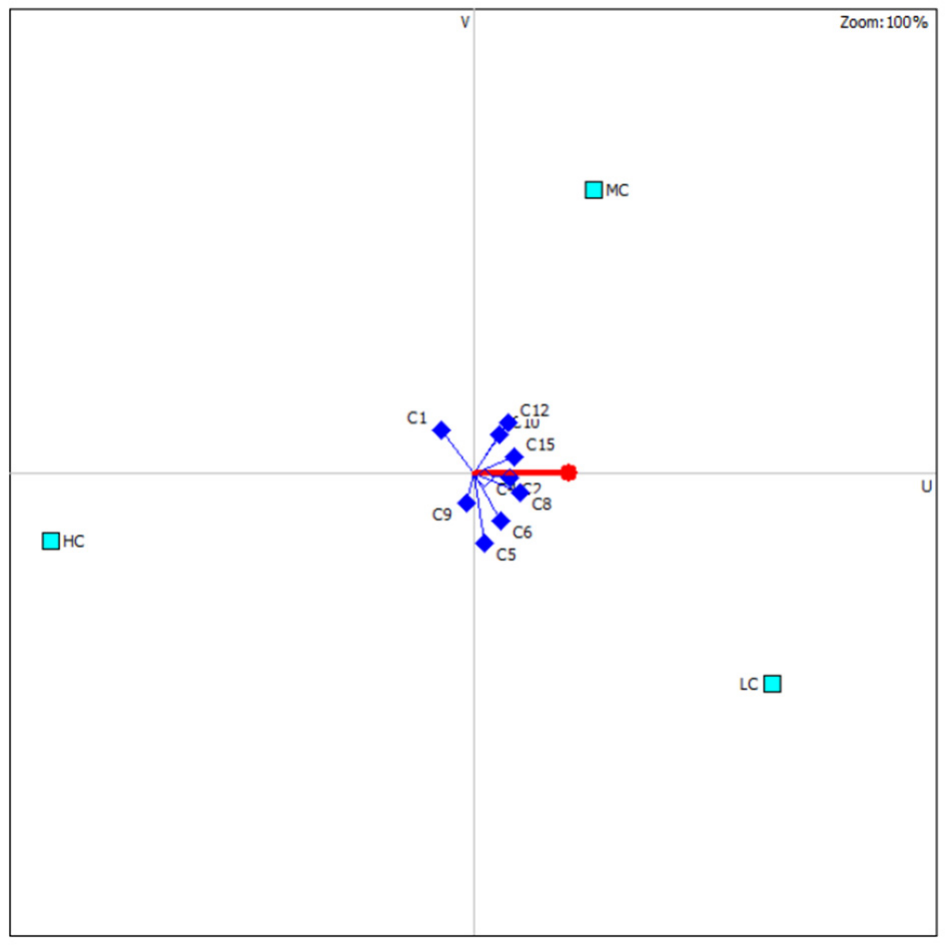
Distribution of criteria for common carp according to the PROMETHEE decision model.

According to all physiological responses of the fish, anesthesia cost, and induction/recovery times, the optimal anesthesia concentration was determined based on the PROMETHEE decision model (Table 5). For common carp, the most suitable anesthesia concentration is 800 μL/L, followed by 1,200 and 1,400 μL/L. According to the distribution of the criteria affecting the ranking, C2, C3, C4, and C8 are the criteria that make LC the preferred concentration (Figure 5). However, C1 in the LC group, which represents induction time, is not at acceptable levels in terms of the applicability of the anesthesia.

**Table 5:** The ranking of different concentration groups in terms of applicability.

**Concentration group**	**ϕ^+^ (*i*)**	**ϕ^−^(*i*)**	**ϕ (*i*)**	**Ranks**
LC	0.5900	0.1625	0.4275	1
MC	0.4281	0.2581	0.1700	2
HC	0.0959	−0.6933	−0.5975	3

LC, 800 μL/L; MC, 1,200 μL/L; HC, 1,400 μL/L.

## 4 Discussion

For an anesthetic agent to be considered applicable, the main factors are that it should not be toxic to animals, should not adversely affect human health during the procedure, and should have suitable induction and recovery times (33). The chemical composition of the essential oil should be investigated, and the anesthetic effects and toxicity of these components must be determined. In the present study, sabinene, which constitutes the largest fraction, is frequently used in the pharmaceutical industry and exhibits antifungal (34) and anti-inflammatory effects (35). On the other hand, α-Pinene and β-Pinene, which are part of the chemical composition of nutmeg essential oil, have been shown to be in essential oil of various plants and produce anesthetic effects in numerous studies (36–41). These components, which bind to the benzodiazepine site of the GABA_A_ receptor complexes in the postsynaptic neurons of the central nervous system, can induce anesthesia in fish via inhibitory postsynaptic potential (IPSP) (42–44). Exposure duration and the concentration of the anesthetic agent can cause stress in the fish and lead to high operational costs (45). Induction time and recovery time are crucial for both minimizing stress on the fish and expediting the process. Past experiences have indicated that induction time should be under 3 min, and recovery time should be under 5 min (46). In the present study, the LC concentration increased the induction time to over 3 min and produced a sedative effect in the fish. In addition, the fish in the MC group were induced and recovered within the appropriate time range. Although the HC group exhibited a rapid induction time, the recovery time occasionally extended to 5 min. Statistical analysis indicates that the MC group is more suitable for both induction and recovery compared to the other groups with longer times. Induction and recovery times may vary depending on the fish species and trophic level (47, 48). In our previous study with Danube sturgeon, it was demonstrated that chamomile oil at lower concentrations induced and recovered the fish in similar time range (7). The use of nutmeg in powder form, as opposed to essential oil, resulted in significantly longer induction and recovery times for common carp (21). This phenomenon can be attributed to the more complete and rapid transfer of anesthetic components from the essential oil into the water. Although a concentration of 300 μL/L of nutmeg seed oil is considered the most suitable for fish transfer, the induction times were still several times higher than acceptable levels (49).

Hematological parameters are among the most rapid indicators of fish response to toxic substances (50). An improperly adjusted concentration of an anesthetic agent can have toxic effects (51). Consequently, fish exposed to the anesthetic may exhibit changes in blood parameters. In the present study, blood parameters were examined over time, showing a continuous increase in WBC, RBC, HGB, and HCT levels during the first 6 h, and returned to near control levels by the eighth hour. This suggests that with a longer observation period, the levels would fully return to control values. Additionally, opercular movement in anesthetized fish slows down (52). This leads to hypoxia, which, in turn, results in elevated catecholamine levels and, ultimately, higher release of RBC, HGB, and HCT from the spleen (53). The results show a similar pattern of HGB, HCT, and RBC in all exposed groups which suggests stress and likely hypoxia caused by gill irritations (epithelial lifting). In aquaculture, the most commonly used natural anesthetic, clove oil, similarly increased blood parameters in fish during the acute phase, with levels returning to normal in subsequent periods (7, 54–57).

Gills are the first organ exposed to water in fish (58). Therefore, although the histological examination of gills may not be fully sufficient to assess the complete stress response of fish in anesthesia studies, it can still be considered an important stress indicator (59, 60). In the current study, hyperplasia was observed in the gill tissues of fish, including the control group, though with lower severity compared to the anesthetized groups. On the other hand, epithelial lifting signs were only seen in the gills of fish subjected to anesthesia. Additionally, necrotic cells were prominently present in the gills of the LC group. Ultimately, as the concentration of the anesthetic increased, the histological changes in the fish also became more pronounced. Hyperplasia and hypertrophy in gill tissues are non-specific responses commonly exhibited by fish to acute and chronic damage (61). These symptoms are generally considered an initial defense mechanism and are classified as mildly reparable (62). In fact, these initial defense responses can be more pronounced in fish anesthetized with clove oil compared to those treated with the synthetic anesthetic agent MS222 (63). Similarly, the use of natural anesthetic agents has been previously reported to induce hyperplasia and epithelial lifting in fish (10, 64).

The alkaline comet assay test is a reliable method for providing information on the genotoxicity of the relevant substance, based on the ICH S2(R1) guideline (65). DNA strand breakage in fish occurs both *in vivo* and *in vitro* in laboratory settings, and it reflects the cells' metabolic activation capacity (66). In the present study, DNA migration was not observed in fish from the control, LC, and MC groups. On the other hand, in the HC group, DNA migration was significantly observed, similar to the positive control. A previously published study has reported conflicting results regarding essential oils and DNA migration in fish, with some studies indicating that essential oils do not induce DNA migration (67), while others have reported that they do (68, 69). Although essential oils are safe at low concentrations, they can exhibit toxic effects when used at high concentrations (51). In previous studies, inconsistencies in the genotoxic effects of essential oils were directly related to the concentrations used. For example, the mutagenic effect of clove oil is dependent on its concentration (70). Clove oil can act as either an antioxidant or a pro-oxidant agent, depending on its concentration, thus potentially serving as a geno-protective agent (71, 72). Eugenol, which is also found in nutmeg oil, has been reported to be genotoxic in various eukaryotic cells, including human cells (73). Although the exact mechanism is not fully understood (68), this genotoxicity has been partially attributed to the oxidative stress induced by eugenol (74). Most essential oils are cytotoxic without being mutagenic and generally do not pose long-term genotoxic risks. Therefore, many of them are reported to be non-carcinogenic and even to enhance apoptosis of tumor cells (75).

The PROMETHEE method, a commonly used multi-criteria decision-making model across various fields, is designed to provide the best option among conflicting criteria (76). Considering the effectiveness of different concentrations of nutmeg essential oil on fish, the optimal concentration has been determined. According to the model, the best score was observed in the LC group, followed by the MC and HC groups, respectively. The criteria that make the LC group highly preferable are C2, C3, C4, and C8, which have the highest weights. However, given that the induction time for anesthesia should be <3 min (46) for the applicability of an anesthetic agent, it is more reasonable to consider LC as a sedation concentration rather than an anesthesia concentration. In fish transport, sedative concentrations are generally used to reduce sensitivity to mechanical stimuli, swimming activity, and stress (77–79). The use of essential oils for sedation reduces stress response, physical damage, and ammonia in water (80–82). Therefore, the LC concentration of nutmeg can be used as a sedative concentration that does not induce stress in fish. Among the other concentrations, the MC group is considered suitable for anesthesia due to its induction and recovery times falling within an acceptable range and its more favorable effects on fish physiology. On the other hand, the HC group presents major limiting factors for suitability due to its histological and genotoxic effects. Additionally, the high cost and extended recovery time negatively impact both the economic feasibility and the practicality of using HC.

## 5 Conclusions

The current study reveals the anesthetic effects of nutmeg essential oil on common carp, a topic previously unexplored. The anesthetic effects of nutmeg essential oil are attributed to α-Pinene and β-Pinene. The induction times for fish in the LC group remained above acceptable levels. As a result of hematological, histological, and genotoxic studies, nutmeg essential oil has emerged as a promising alternative to synthetic anesthetic agents. However, the most concerning aspect of the study is the regions where nutmeg oil is distributed, which contributes to its high cost. Since nutmeg is predominantly a plant found in far east countries, the availability and applicability of this anesthetic agent in countries like India, Pakistan, and Indonesia are of particular importance. In future studies, it is essential to investigate stress parameters such as plasma cortisol and glucose levels to further validate these findings.

## Data Availability

The original contributions presented in the study are included in the article/Supplementary material, further inquiries can be directed to the corresponding author.
