# Supplementary material for: A new herbal anesthetic agent for common carp (Cyprinus carpio) sedation and anesthesia: nutmeg (Myristica fragrans) essential oil
Source: Front Vet Sci. 2024 Oct 14;11:1477357. doi: 10.3389/fvets.2024.1477357 (PMC11514786; doi:10.3389/fvets.2024.1477357)
Supplement: Supplementary file 1 [file Table_1.docx]

**Supplementary Material**

**A new herbal anesthetic agent for common carp (*Cyprinus carpio*) sedation: Nutmeg (*Myristica fragrans*)**

**SM1**

*Selection and ranking of best alternatives by PROMETHEE analysis*

The best concentration of nutmeg essential oil for three different fish species was determined with the PROMETHEE decision model (Visual PROMETHEE 1.1.0.0). After the evaluation criteria and the weight values of these criteria were determined, a decision matrix was created. PROMETHEE allows decision makers to make a specific choice in terms of an evaluation factor or to limit the evaluation factor to values they specify. PROMETHEE decision model is a method that reaches results in a total of seven steps.

Step one: The data matrix is prepared using equations 1, 2, and 3. Criterion weights are determined for *k* number criteria (*k*=10 in the current study).

$w= w_{1},w_{2},\ldots,w_{k}$ (1)

$w:criteria weight$

$c= f_{1},f_{2},\ldots,f_{k}$ (2)

$c:criteria weight$ and $f:function$

$S=(A,B,C,\ldots)$ (3)

$$S:decision alternatives$$

Step two: Preference functions for the criteria are determined according to Equation 4 (linear preference function) and Equation 5 (usual preference function).

$p\left( d \right)=\left\{ \begin{matrix} 0 & d\leq q \\ (d-p)/(p-q) & q<d\leq p \\ 1 & d>p \end{matrix}(d-p)/(p-q) \right.$ (4)

$$q:indifference value$$

$$p:sufficient biggest difference$$

$$q:difference between two decision alternatives$$

$p\left( d \right)=\left\{ \begin{matrix} 0 & d\leq0 \\ 1 & d>0 \end{matrix} \right.$ (5)

Step three: The common preference function for decision alternatives “x” and “y” is calculated with Equation 6.

$p\left( x,y \right)=\left\{ \begin{matrix} 0 & f(x)\leq f(y) \\ p[f\left( x \right)-f\left( y \right)] & f(x)>f(y) \end{matrix} \right.$ (6)

According to Equation 6, it is determined whether the evaluation factor is maximization or minimization.

Step four: The preference index of “x” and “y” decision options evaluated according to the k-number criterion was calculated using Equation 7.

$\pi\left( x,y \right)=\sum_{i=1}^{K} w_{i}P_{i}(x,y)$ (7)

Step five: Determining positive φ^+^ and negative φ^−^ advantages for alternatives with Equations 8 and 9.

$\varphi^{+}\left( x \right)=\frac{1}{n-1}\sum\pi(x,y)$ (8)

$\varphi^{-}\left( x \right)=\frac{1}{n-1}\sum\pi(y,x)$ (9)

Step six: Partial priorities are determined with PROMETHEE I. Equations 10 and 11 show the difference between “x” and “y” decision alternatives. If any of the following conditions occur, decision option “x” is indistinguishable from decision option “y”.

$\varphi^{+}\left( x \right)=\varphi^{+}\left( y \right)$ (10)

$\varphi^{-}\left( x \right)=\varphi^{-}\left( y \right)$ (11)

Similar to the example below, if any of the conditions in Equation 12, 13,14 occur, the “x” decision option is superior to the “y” decision option.

$\varphi^{+}\left( x \right)>\varphi^{+}\left( y \right) and \varphi^{-}\left( x \right)<\varphi^{-}\left( y \right)$ (12)

$\varphi^{+}\left( x \right)>\varphi^{+}\left( y \right) and \varphi^{-}\left( x \right)=\varphi^{-}\left( y \right)$ (13)

$\varphi^{-}\left( x \right)<\varphi^{-}\left( y \right) and$ $\varphi^{+}\left( x \right)=\varphi^{+}\left( y \right)$ (14)

In the condition where decision alternative "x" cannot be compared with decision alternative "y", equations 15 and 16 are used.

$\varphi^{+}\left( x \right)>\varphi^{+}\left( y \right) and \varphi^{-}\left( x \right)>\varphi^{-}\left( y \right)$ (15)

$\varphi^{+}\left( x \right)<\varphi^{+}\left( y \right) and \varphi^{-}\left( x \right)<\varphi^{-}\left( y \right)$ (16)

Step seven: The ranking of decision options is performed with PROMETHEE II. The exact priorities of the decision options are determined by Equation 17. All calculated priority values are sorted from high priority to low priority. Thus, all decision options are evaluated in a similar way and a complete ranking is obtained.

$\varphi\left( x \right)=\varphi^{+}\left( x \right)-\varphi^{-}\left( x \right)$ (17)

The decisions given in equations 18 and 19 can be reached according to the full priority value calculated from the “x” and “y” decision alternatives.

$\varphi\left( x \right)>\varphi\left( y \right)$ (18)

Decision alternative “x” is superior.

$\varphi\left( x \right)=\varphi\left( y \right)$ (18)

Decision alternatives “x” and “y” are not superior.
